# The impact of narcissistic personality traits on social media interaction and body image: individual and team athletes

**DOI:** 10.3389/fpsyg.2026.1848790

**Published:** 2026-05-22

**Authors:** Kübra Kiliç, Burcu Sıla Sezer, İbrahim Aydinli, Ömer Faruk Tutar, Yakup Kiliç

**Affiliations:** 1 Ministry of Health, Elazığ, Türkiye; 2 Faculty of Sports Sciences, Bingöl University, Bingöl, Türkiye; 3 Faculty of Sports Sciences, Kocaeli University, Kocaeli, Türkiye; 4 Faculty of Sports Sciences, Munzur University, Tunceli, Türkiye; 5 Faculty of Sports Sciences, Fırat University, Elazığ, Türkiye

**Keywords:** body image, individual sports, narcissistic, social media, team sports

## Abstract

**Introduction:**

The aim of this study is to examine the effect of narcissistic personality traits on body image through social media interaction and to comparatively evaluate these relationships in the context of athletes engaged in individual and team sports.

**Methods:**

The research group consisted of a total of 389 athletes, namely 158 female (age = 21.53 ± 3.16; years of sports experiences = 5.56 ± 4.17) and 231 male (age = 23.00 ± 5.09; years of sports experience = 8.28 ± 5.44) athletes, all residing in the Eastern Anatolia region and having a sports background. A personal information form, the “Narcissism on Social Media Scale,” and the “Body Image Scale” were used as data collection tools. Analysis of the obtained data revealed a normal distribution; therefore, t-tests were used to compare two groups, ANOVA for comparisons of two variables, Pearson Correlation to evaluate relationships between parameters, and regression analysis to determine the effect.

**Results:**

As a result of the analyses, no significant difference was observed in terms of the type of sport variable. The study found that, among athletes engaged in individual sports, body image levels were higher in males compared to females and, in individual athletes, the narcissistic rivalry dimension was more prevalent in athletes who used social media for 5 h or more compared to those using it for less than 1 h. Multiple correlation analysis revealed no significant relationship between scale dimensions and years of sports experience. Regression analysis showed that body image level had a low but significant effect on narcissistic admiration and narcissistic rivalry.

**Conclusion:**

In conclusion, it was determined that athletes in individual sports are sensitive to demographic variables, and that the duration of social media use, in particular, is a factor that increases narcissistic competitive tendencies. The fact that years of sports experience and type of sport did not make a difference suggests that the dimensions used are shaped more by current psychosocial interactions. Indeed, the regression analysis also shows that the relationship between body image and narcissistic admiration and competition points to a weak but significant psychological interaction network.

## Introduction

In the modern era, the desire to establish your sense of self is not merely a need but a necessity. The desire to feel important, to idealize oneself, and have high expectations emerge from the turmoil of the modern age and have profound effects on people (Kristinsdottir et al., 2021; Miller et al., 2021). In this context, the effort to make the self unique, special, and superior is rooted in a psychological framework that can be explained by the concept of narcissism (Akdeniz et al., 2022). Initially conceptualized as a one-dimensional structure (Gormley and Lopez, 2010; Barelds et al., 2017; Chin et al., 2017; Ganster et al., 2025), this tendency has been examined in two different dimensions over time (Cain et al., 2008; Miller et al., 2011). These dimensions are grandiose and vulnerable narcissism. Grandiose narcissism is associated with attention-seeking, expectation of privilege, perception of superiority, and feelings of dominance (Pincus and Lukowitsky, 2010; Kaufman et al., 2020); while vulnerable narcissism is associated with behaviors such as low self-esteem, expectation of approval, anger, anxiety, and shame (Rohmann et al., 2019). This disorder, characterized by a pervasive pattern of grandiosity, a need for admiration, a desire to pursue status, and a lack of empathy (Weinberg and Ronningstam, 2022; Zeigler-Hill and Dehaghi, 2023; Jacobsen et al., 2026), is also associated with many negative behaviors such as manipulation and aggression in conflict situations (Tazegül, 2011; Tazegül and Güven, 2015; Altınok and Kılıç, 2020; Mohay et al., 2025). In the context of sports, these two concepts are characterized by varying behavioral patterns. In grandiose narcissism, the athlete is less concerned about competition and believes they can achieve first place; conversely, in vulnerable narcissism, sensitivity to criticism and a tendency toward comparison are prominent (Öner, 2023). However, narcissism cannot always be classified as a disorder; it can also be explained as a natural personality trait that we all possess and that expresses an individual’s love for themselves.

From an athlete’s perspective, the ability to enjoy their activities, feel pride in their successes, and balance feelings of shame in their failures is considered harmless narcissism (Gülmez, 2009). This situation may stem from athletes’ efforts to achieve success and goals (Şahinler et al., 2021). Sport, by its very nature, aims for the holistic development of performance (Yalcin et al., 2026). In this context, athletes are evaluated not only on their physical capacity but also on their appearance during competition and performance processes, which makes their self-presentation critical (Suffolk, 2014; Kaya and Uludağ, 2025).

At this point, the distinction between healthy and pathological manifestations of narcissism raises the question of the sources from which an individual draws their self-evaluation. Body image, a significant component of the self, is directly related to narcissism. Fueled by attention, approval, and admiration, body image is the mental image of one’s body (Önal et al., 2019). This concept, evaluated in two stages, is, in the first stage, the individual’s subjective beliefs about their appearance and, in the second stage, the investment they make in their body image resulting from evaluations (Morrison et al., 2004). For athletes, body image is considered not only an esthetic matter (Uzun, 2025) but also a determinant of performance (Burgess et al., 2006). However, with the development of mass media, this factor has transformed into an esthetically focused structure (Krane et al., 2001). However, body image is shaped not only by the individual’s mind but also by their sociological environment, physical appearance, mass media, and cultural attitudes (Erdoğan and Tütüncü, 2015). In this sense, social media has a significant impact on body image (Oktan and Şahin, 2010).

Content shared on social media provides the opportunity to be more visible thanks to the like button (Arslan and Taydaş, 2023). Therefore, individuals place more importance on how their bodies are perceived in terms of external appearance on social media (Çetinkaya et al., 2022). Because they provide opportunities for both self-presentation and attention, they are also ideal platforms for narcissistic self-regulation (Forest and Wood, 2012; Alemdar et al., 2017; Balcı and Sarıtaş, 2019). Indeed, it is known that narcissistic individuals mostly try to construct their sense of self in the social sphere (Ong et al., 2011; Boursier et al., 2020). All of this constitutes one aspect of how social media has become a tool for self-presentation for individuals (Zheng et al., 2020).

Researchers have also supported the positive correlation between social media use and narcissism (Carpenter, 2012; Oğuz, 2016; Akpınar and Karakoç, 2022; Çimke and Gürkan, 2023). The question is whether social media affects narcissism or, conversely, whether narcissism affects social media use (Kurunç and Şahin, 2023). When evaluated within the context of sports, it is thought that this relationship may differ depending on the structural characteristics of the sport. In particular, those engaged in individual sports branches, due to their direct involvement with themselves, tend to exhibit more intense social media communication behaviors (Karagün and Tapşın, 2024); athletes involved in team sports, however, spend less time in social environments or stay away from social media accounts due to spending time with their teammates (Yazan et al., 2024). It has also been determined that athletes with narcissistic traits tend to take on more roles within teams (Woodman et al., 2011). Although it is stated that narcissistic individuals are more successful in individual sports, it is known that they also gravitate toward team sports such as football and basketball due to the social appreciation and visibility they offer (Yaşar and Sunay, 2017). Therefore, it is considered that the relationship between social media use, narcissism, and body image may be shaped through different dynamics between individual and team athletes.

In this context, the role of social media on narcissism and body image has become more significant with the development of technology and the changing world. However, a review of the existing literature reveals that the relationships between narcissism, social media, and body image have mostly been studied in the general population, while these relationships have not been adequately addressed in athlete samples. Furthermore, there are a limited number of studies on how the relationships of these parameters differ between individual and team athletes. This situation creates a significant gap in explaining the interaction of these variables in the context of sports. The aim of this study is to examine the effect of narcissistic personality traits on body image through social media interaction and to evaluate these relationships comparatively in the context of athletes engaged in individual and team sports. In this context, the following hypotheses were formulated:

*H1*: Narcissistic personality traits are significantly related to the level of social media interaction and body image.

*H2*: The effect of narcissistic personality traits on social media interaction and body image differs significantly between individual and team athletes.

## Methods

### Research model

Research data was collected online using Google Forms. Athletes were asked specific questions via a survey form, and data were obtained from the answers to these questions. In this context, a relational survey model was used in the study sample. This model is used to determine the relationships between the variables examined in the research and the degree to which these variables coexist (Karasar, 2018). This model, in accordance with the research objective, provided results aimed at understanding the characteristics of the athletes and the relationships between these characteristics. In this study, the relationship between the narcissistic personality traits of individual and team athletes and their social media interaction and body image level was statistically examined.

### Study group

A total of 389 athletes (40.6% female and 59.4% male athletes) who participated in individual and team sports took part in this study. The average age of the athletes was 22.40 ± 4.46 years. The average age of the female athletes (*n* = 158) was 21.53 ± 3.16 years; their average years of sports experience was 5.56 ± 4.17 years; the average age of the male athletes (*n* = 231) was 23.00 ± 5.09 years; and their average years of sports experience was 8.28 ± 5.44 years. Participants were included in the study according to the following criteria: (i) actively involved in sports (individual or team) for at least 1 year, (ii) being over 18 years of age, and (iii) voluntarily participating in the study. Individuals who did not meet these criteria were excluded from the analysis.

### Sample size estimation

The sample group of the research consisted of individuals living in the Eastern Anatolia region with an athletic background. An *a priori* power analysis was conducted using G*Power 3.1 (Hein-rich-HeineUniversity Düsseldorf, Düsseldorf, Germany) to determine the required sample size. The analysis was performed within the t-test family, specifically using the means: difference between two independent means (two groups) model. The effect size (Cohen’s d) was set at 0.50, the significance level (*α*) at 0.05, and a one-tailed test was specified. The desired power statistical (1 − *β*) was set at 0.93.

The results indicated that a minimum of 70 participants per group was required to achieve the specified power level, resulting in a total sample size of at least 140 participants. These findings suggest that the study is adequately powered to detect small-to-moderate effect sizes with high statistical sensitivity. Given the available data records, 389 participants were included in the study, and all statistical analyzes were conducted on these samples. Therefore, the sample size in this study was deemed sufficient.

### Data collection tools

Personal Information Form: To obtain descriptive data of the athletes, the researchers asked the athletes in the study specific questions. Through these questions, researchers attempted to ascertain information about the athletes such as gender, age, years of sports experience, daily social media usage time, and type of sport. This data was used to define and analyze participants’ characteristics, in accordance with the research objective.

#### Body image scale

The Body Image Scale was developed by Secord and Jourard (1953). It was adapted into Turkish by Hovardaoğlu (1993). Consisting of 40 items in total, the scale has a unidimensional structure. The scale is a 5-point Likert scale (1 = Very much like, 5 = Not at all like) and does not contain reverse items. An increase in the scores obtained from the scale indicates increased body satisfaction among participants. In the adapted study, internal consistency values were reported. For the body image dimension, *α* = 0.91 was determined. These values indicate an acceptably high level of internal consistency for the scale dimension. In the study conducted, *α* = 0.97 was determined for the body image dimension.

#### Narcissism on social media scale

The Narcissism on Social Media Scale was developed by Akdeniz et al. (2022). This scale consists of 16 items and is evaluated in two sub-dimensions. These sub-dimensions are sub-dimensions of Narcissistic Admiration and Narcissistic Competition. The scale is a 5-point Likert type (1 = Not suitable at all, 2 = Not very suitable, 3 = Undecided, 4 = Suitable, 5 = Completely suitable). Internal consistency values were reported in the study. For the Narcissistic Admiration dimension, *α* = 0.80 was determined, and for the Narcissistic Competition dimension, *α* = 0.76 was determined. Similarly, in the study conducted, *α* = 0.84 was determined for the Narcissistic Admiration sub-dimension and *α* = 0.83 for the Narcissistic Competition sub-dimension.

### Statistical analysis

The data obtained in the study were analyzed using the SPSS 27 software package. In order to examine the normality of the data, skewness and kurtosis values were calculated and evaluated using the ±1.5 reference range suggested by Tabachnick and Fidell (2013) (Table 1). According to this evaluation, it was assumed that the data were normally distributed. Independent samples t-test was used for variables such as gender and type of sport. An ANOVA test was performed for the social media usage duration variable, Pearson correlation analysis was performed to examine the relationship between years of sport, the body image scale, and the narcissism on social media scale dimensions, and regression analysis was performed to determine the effect between scale dimensions. The significance level was accepted as *p* < 0.05 (Tables 2–6).

**Table 1:** Analysis of mean, standard deviation, skewness, and kurtosis values of the body image and the narcissism on social media scales.

Scale	Dimensions	X̄	SD	Skewness	Kurtosis
Body image scale	Body image total	96.26	39.77	0.531	−0.298
Narcissism on social media	Narcissistic Admiration	29.76	7.39	−0.545	0.452
	Narcissistic competition	18.52	6.39	0.219	−0.444

X̄, Mean; SD, Standard deviation.

**Table 2:** Evaluation of the body image scale and social media narcissism scale dimensions of individual and team sports athletes according to gender variable.

Type of sports	Scale	Dimensions	Gender	*n*	X̄	SD	*t*	*p*
Individual sports	Body image	Body image total	Female	87	86.18	34.03	−3.150	0.002*
			Male	113	104.23	44.30		
	Narcissism on social media	Narcissistic admiration	Female	87	30.71	6.32	0.915	0.361
			Male	113	29.76	7.84		
		Narcissistic competition	Female	87	17.59	5.96	−1.154	0.250
			Male	113	18.59	6.11		
Team sports	Body image	Body image total	Female	71	90.52	33.97	−1.564	0.120
			Male	118	99.52	40.72		
	Narcissism on social media	Narcissistic admiration	Female	71	29.73	7.36	0.584	0.560
			Male	118	29.06	7.69		
		Narcissistic competition	Female	71	18.87	6.39	−0.067	0.947
			Male	118	18.94	6.94		

*p < 0.05 bold values indicate levels of statistical significance.

**Table 3:** Evaluation of body image and social media narcissism dimensions according to sport type variable.

Scale	Dimensions	Type of sports	*n*	*X*	SD	*t*	*p*
Body image	Body image total	Individual	200	96.38	41.05	0.059	0.953
		Team	189	96.14	38.48		
Narcissism on social media	Narcissistic admiration	Individual	200	30.18	7.22	1.151	0.250
		Team	189	29.31	7.55		
	Narcissistic competition	Individual	200	18.16	6.05	−1.166	0.244
		Team	189	18.91	6.72		

**Table 4:** Evaluation of body image scale and social media narcissism scale dimensions of individual and team sport athletes according to daily social media usage time.

Type of sports	Scale	Dimensions	Daily social media usage time	*n*	X̄	SD	*F*	*p*	Tukey
Individual sports	Body image	Body Image Total	1 h or less^a^	18	110.55	39.43	1.242	0.296	–
			1–2 hours^b^	47	99.74	42.01			
			3–4 hours^c^	88	95.14	42.02			
			5 h or more^d^	47	89.89	38.40			
	Narcissism on social media	Narcissistic Admiration	1 h or less^a^	18	27.38	10.08	2.337	0.075	–
			1–2 hours^b^	47	28.70	8.00			
			3–4 hours^c^	88	31.26	6.22			
			5 h or more^d^	47	30.70	6.55			
		Narcissistic competition	1 h or less^a^	18	16.88	6.58	4.959	0.002*	**d > a**
			1–2 hours^b^	47	15.82	5.41			
			3–4 hours^c^	88	18.51	5.70			
			5 h or more^d^	47	20.31	6.35			
Team sports	Body image	Body image total	1 h or less^a^	11	85.36	37.90	1.605	0.190	
			1–2 hours^b^	41	101.36	39.10			
			3–4 hours^c^	80	100.33	37.16			
			5 h or more^d^	57	88.57	39.36			
	Narcissism on social media	Narcissistic Admiration	1 h or less^a^	11	28.00	11.79	0.415	0.743	
			1–2 hours^b^	41	29.73	7.86			
			3–4 hours^c^	80	28.81	6.41			
			5 h or more^d^	57	29.98	7.96			
		Narcissistic Competition	1 h or less^a^	11	18.27	9.54	0.321	0.811	
			1–2 hours^b^	41	18.85	6.97			
			3–4 hours^c^	80	18.53	6.06			
			5 h or more^d^	57	19.61	6.93			

*p < 0.05 bold values indicate levels of statistical significance.

**Table 5:** Pearson correlation results of body image and social media narcissism dimensions according to years of sports experience.

Type of sports		Variables		(1)	(2)	(3)	(4)
Individual sports		Years of sports experience (1)	r	1			
			p				
	Body image	Body image total (2)	r	0.058	1		
			p	0.415			
	Narcissism on social media	Narcissistic admiration (3)	r	0.058	−0.055	1	
			p	0.415	0.443		
		Narcissistic competition (4)	r	0.061	0.070	0.460	1
			p	0.393	0.322	0.000**	
Team sports		Years of sports experience (1)	r	1			
			p				
	Body image	Body image total (2)	r	−0.014	1		
			p	0.852			
	Narcissism on social media	Narcissistic admiration (3)	r	0.089	−0.123	1	
			p	0.223	0.092		
		Narcissistic competition (4)	r	−0.049	0.020	0.595	1
			p	0.506	0.786	0.000**	

**n, 389; *p* < 0.01.

**Table 6:** Results of regression analysis predicting the dependent variable.

Variables	*β*	*t*	*p*	*R* ^2^	_Adj_ *R* ^2^	F	*f* ^2^	sr^2^
Constant		12.469	0.001*	0.019	0.014	3.712	0.02	
Narcissistic admiration	−0.153	−2.575	0.010*					0.017
Narcissistic competition	0.125	2.110	0.036*					0.011

*p < 0.05 bold values indicate levels of statistical significance; *β* = standardized regression coefficient; _Adj_. *R*^2^ = adjusted coefficient of determination; *f*^2^ = Cohen *f*^2^; sr^2^ = Squared semi-partial correlation.

When the skewness-kurtosis values of the body image and social media narcissism scale dimensions were examined, it was found that the skewness values ranged from −0.545 to 0.531, and the kurtosis values ranged from −0.298 to 0.452.

## Results

A statistically significant difference was found in the body image levels of participants in individual sports based on gender. Accordingly, it was determined that male participants had higher body image levels than female participants.

When examining the dimensions of the social media narcissism scale, no significant difference was found in the narcissistic rivalry and admiration dimensions. For participants in team sports, no significant difference was found in the total body image scale and the narcissistic admiration and rivalry dimensions of the social media narcissism scale based on gender.

No significant differences were found in the total body image scale and the narcissistic admiration and competition dimensions of the social media narcissism scale based on the type of sport participated in.

When examining the dimensions of the narcissism on social media scale among participants engaged in individual sports, a statistically significant difference was found in the narcissistic competition dimension when compared according to the variable of daily social media usage time. Accordingly, it was determined that participants who had a daily social media usage time of 5 h or more had higher levels of competition compared to those who used it for less than 1 h. However, when the total dimensions of the Narcissistic Admiration and Body Image scales were examined, no significant difference was found.

For participants engaged in team sports, no significant difference was found in the total body image scale and the narcissistic admiration and competition dimensions of the social media narcissism scale according to the variable of social media usage time.

According to the results of the multiple correlation analysis, no significant relationship was found between years of sports experience and scale dimensions in both individual and team athlete samples.

Furthermore, no significant relationship was found in the total dimension of the body image scale and the narcissistic admiration and competition dimensions of the social media narcissism scale in both individual and team athlete samples.

Multiple linear regression analysis revealed that the model is statistically significant overall (*F* = 3.712, *p* = 0.025). Although the model’s explanatory power is low, when the independent variables are considered together, it explains approximately 1.9% of the variance in body image (*R*^2^ = 0.019, _Adj_*R*^2^ = 0.014). When the effect size is examined, it is seen that the model has a small effect (*f*^2^ ≈ 0.02).

When examined on a variable basis, it was determined that the narcissistic admiration variable has a negative and significant effect on body image (*β* = −0.153, *t* = −2.575, *p* = 0.010, sr^2^ = 0.017). This result shows that, as the level of narcissistic admiration increases, there is a tendency for individuals’ body image scores to decrease. In contrast, the narcissistic competition variable was found to have a positive and significant effect on body image (*β* = 0.125, *t* = 2.110, *p* = 0.036, sr^2^ = 0.011).

This finding suggests that individuals with a high tendency toward competition may have relatively higher body image. However, it appears that both variables have low unique explanatory power, and the overall explanatory power of the model remains limited.

## Discussion

This study examined the extent to which narcissistic personality traits in individual and team sports participants are influenced by social media interaction and body image. The findings were evaluated in relation to gender, years of sports experience, type of sport, duration of social media use, and the dimensions of the scales used. The findings reveal that body image is not associated with narcissistic admiration and competition. However, regression analyses show that body image has a low-level significant effect on narcissistic admiration and competition. Accordingly, it can be suggested that the level of body image is not a strong determinant alone in the development of narcissistic personality traits but rather plays a contributing role. The low level of influence may be related to the fact that narcissism and body image tendencies are a phenomenon observable in many different factors related to sports (Öner, 2023). The standardized perception of beauty in social media and digital communication channels can lead to a detachment from reality perception and distortions in body image in narcissistic individuals (Önal, 2025). Indeed, studies in the literature have shown that athletes’ body image can have significant effects on their level of narcissism (Ateş et al., 2023; Szymczak et al., 2023; Haider et al., 2025; Kaya and Uludağlı, 2025). In this context, it can be considered that, as the level of body image increases, athletes may tend to display themselves (narcissistic admiration) and compete with individuals they encounter on social media (narcissistic competition). In this context, the influence of body image on narcissistic tendencies can be explained not directly but rather through indirect psychological processes. Particularly with the increased use of social media, individuals constantly compare their physical appearance to others and develop self-evaluation based on external validation (Yorulmaz and Kurutçu, 2019; Tayhan, 2023). This process causes body image to function as a mediating mechanism that triggers narcissistic admiration (self-display and the pursuit of approval) and narcissistic competition (comparison and establishing superiority) (Balcı and Sarıtaş, 2019; Güler, 2025). Therefore, body image, rather than being the sole determining factor in the emergence of narcissism, can exert its influence through processes such as social media interaction, self-presentation, and the need for validation. On the other hand, contrary to the current findings, Zihidro and Aner-Aktan (2024) reported that body image and narcissistic personality roles do not interact. This inconsistency may stem from the contextual and variable nature of body image and the fact that dimensions of narcissism with different functions, such as admiration and competition, assume different roles in self-regulation processes.

When the difference between the type of sport (individual/team) and body image and social media narcissism was examined, no significant difference was found. This result suggests that psychosocial factors may be more decisive than the multifaceted nature of sports in determining body image and the level of social media narcissism. While the performance and visibility of individual athletes are linked to their individuality, group dynamics and social communication are paramount in team sports (Karagün and Tapşın, 2024; Yazan et al., 2024). Initially, it might be assumed that social media narcissism and body image could affect both of these sport types. However, the study found that the predicted difference did not exist, and this is due to the indirect impact of the widespread use of mass platforms in the context of sports (Giancola et al., 2025). This result indicates that psychosocial factors may be more decisive in determining body image and social media narcissism levels in individuals, outside of the multidimensional nature of sports. In parallel, studies in the literature have found no significant differences in narcissistic personality and body image levels between participants in team and individual sports (Cankurtaran and Berısha, 2021; Caniklitemel and Ağralı Ermiş, 2024). This finding does not support the expectation that sport type is linked to narcissism and body image. Contrary to the current findings, some studies have reported that individual and team sports are linked to narcissistic personality (Tazegül, 2013) and body image levels (Güçlü and Yentürk, 2008). Indeed, it has been frequently mentioned in the literature that individual and team sports even differ in the personality traits they contribute to their athletes (Yaşar and Sunay, 2017; Keya, 2022). The literature also suggests that the inconsistencies in sport-specific differences may be related to the internalization of psychosocial and body ideals independently within the context of sports.

When comparing the average social media narcissistic personality scores of athletes in terms of gender, no significant difference was found. However, when comparing average body image scores, it was determined that male participants in individual sports obtained significantly higher scores than female participants. Therefore, it can be said that male athletes have a higher level of body image than female athletes. It is thought that the main reason for this may be that male athletes place more importance on body evaluations focused on physical fitness and muscular appearance. According to sociocultural theory, the muscular body image, which is considered ideal among male athletes due to the influence of mass media, is affected (Thompson and Stice, 2001). In a similar study, Hart et al. (2008) stated that the reason why men’s social appearance concerns are higher than women’s is due to men’s desire to appear stronger and more muscular. However, there are also studies that claim the opposite. In their study, Caniklitemel and Ağralı Ermiş (2024) stated that female participants placed a higher degree of importance on their body image and that this was related to the fact that female participants pay more attention to their bodies and receive positive feedback from their environment.

No significant relationship was found between the years of sports experience and the scale dimensions of individual and team sports athletes. This may indicate that the years of sports experience are not a determinant of psychological structures related to narcissism and body image. Especially with the widespread use of mass media, sports experience may no longer play a decisive role in more complex psychosocial structures such as narcissism and body image. Although the literature frequently mentions that the years of sports experience play an active role in self-efficacy, performance, and psychological processes (Bukay and Şahlar, 2025), it can be said that this effect is not valid for every variable. Indeed, parallel to the findings of this study, there are studies indicating that the duration of experience is not related to body image and narcissistic personality traits (Köse et al., 2016; Caniklitemel and Ağralı-Ermiş, 2024; Özsaydı et al., 2025). This situation can be attributed to the fact that sports experience alone is not sufficient when it comes to structures such as social media interaction, narcissism, or body image, which are shaped more by the interaction of an individual’s environmental and personality traits.

When examining the dimensions of the narcissism scale on social media use among participants in individual sports in terms of the variable of social media usage time, a statistically significant difference was determined in the narcissistic competition dimension. Since narcissistic competition is a structure in which individuals strive for superiority, engage in hostile self-defense, and devalue their opponent, it reflects a psychological tendency quite compatible with the nature of social media (Back et al., 2013). Social media platforms provide an environment where participants can constantly compare themselves to others and showcase their performance and physical appearance (Alemdar et al., 2017; Çetinkaya et al., 2022). Therefore, since performance in individual sports is directly based on individual success, the increased use of social media may strengthen athletes’ tendency to showcase themselves and compare themselves to others. Studies supporting this have also reported that narcissistic personality traits increase with increased social media usage time (Güler, 2025; Kaya, 2025).

## Limitations

This study was limited to students studying at Faculties of Sports Sciences in the Eastern Anatolia region, exhibiting specific age and amateur athlete profiles. The data obtained in this study were evaluated within the framework of a cross-sectional research design. Therefore, it only reveals the relationships between variables and cannot be interpreted in terms of causality. The data were collected through self-report. This situation may lead to measurement errors such as individuals’ socially desirable status and the possibility of different self-evaluations. In addition, the fact that social media use was evaluated only with the variable of duration is a significant limitation. This limits a deeper understanding of the psychological effects of social media. Finally, potential mediating or moderating variables (self-esteem, social comparison tendency, psychological well-being, etc.) were not included in the study.

## Conclusion

This study reveals that the relationships between body image and social media-related narcissistic personality traits in athletes are complex and multifaceted. Sport type is not a determining factor in narcissistic personality traits and body image levels, but gender and social media usage duration are particularly influential in narcissistic competition. However, the presence of a significant but limited effect of body image on narcissistic admiration and narcissistic competition is noteworthy. Based on these findings, it is suggested that the effects of social media should be considered in understanding psychosocial processes in athletes, and that body image plays a related but indirect role in relation to narcissistic personality traits.

## Recommendations

Longitudinal and experimental research designs are recommended to more clearly reveal the relationships between variables. Examining social media use not only in terms of duration but also qualitatively (content type, purpose, format, etc.) will broaden the scope of studies. In future research, it is recommended that psychosocial variables such as social comparison and self-esteem be included in the model in order to more comprehensively explain the processes associated with social media narcissism and body image perception. Considering the high levels of narcissistic competition observed in individual athletes, practitioners and sports psychologists can create individualized support programs by taking into account athletes’ social media usage habits and psychosocial characteristics. It is recommended that sports experience be considered not only in terms of duration but also in terms of qualitative dimensions such as training frequency, level of competition, and psychological skills training. In general, it is suggested that future studies be expanded to include different age groups, sports branches, and cultural contexts.

## Data Availability

The original contributions presented in the study are included in the article/supplementary material, further inquiries can be directed to the corresponding author/s.
